# Drug–Cannabinoid Interactions in Selected Therapeutics for Symptoms Associated with Epilepsy, Autism Spectrum Disorder, Cancer, Multiple Sclerosis, and Pain

**DOI:** 10.3390/ph17050613

**Published:** 2024-05-10

**Authors:** Maria G. Campos, Maria China, Mariana Cláudio, Miguel Capinha, Rita Torres, Simão Oliveira, Ana Fortuna

**Affiliations:** 1 Observatory of Drug-Herb Interactions, Faculty of Pharmacy, University of Coimbra, Health Science Campus, Azinhaga Santa Comba, 3000-548 Coimbra, Portugal; mariasgchina@sapo.pt (M.C.); simaumsamp@icloud.com (S.O.); 2 Coimbra Chemistry Centre (CQC, FCT Unit 313) (FCTUC), University of Coimbra, Rua Larga, 3004-531 Coimbra, Portugal; 3 Laboratory of Pharmacology and Pharmaceutical Care, Faculty of Pharmacy, University of Coimbra, 3000-548 Coimbra, Portugal; 4 CIBIT—Coimbra Institute for Biomedical Imaging and Translational Research, ICNAS, University of Coimbra, 3000-548 Coimbra, Portugal

**Keywords:** cannabinoids, tetrahydrocannabinol, CBD, drug–drug interactions, cytochrome P450, epilepsy, cancer, autism spectrum disorder

## Abstract

Clinical practice entails a translation of research that assists in the use of scientific data and therapeutic evidence for the benefit of the patient. This review critically summarizes the potential impact of cannabinoids in conjunction with other drugs when associated with treatments for epilepsy, autism spectrum disorder, cancer, multiple sclerosis, and chronic pain. In these associations, potential drug interactions may occur and alter the predicted clinical results. Therefore, the potential for drug interactions must always be assessed to avoid therapeutic failures and/or increased side effects. Some effects may be additive, synergistic, or antagonistic, but changes in absorption, distribution, metabolism, particularly through cytochrome P450 (CYP) isoenzymes (e.g., CYP2C9 and CYP3A4), and excretion may also occur. For example, the combination of cannabis-derived compounds and the antifungal drug ketoconazole, a CYP3A4 inhibitor, increases the plasma concentration of Δ-9-tetrahydrocannabinol (THC) and cannabidiol (CBD). In contrast, rifampicin, a CYP3A4 inducer, stands out for reducing plasma THC levels by approximately 20–40% and 50% to 60% for CBD. Other CYP3A4 inhibitors and inducers are likely to have a similar effect on plasma concentrations if co-administered. Pharmacokinetic interactions with anticonvulsant medications have also been reported, as have pharmacodynamic interactions between cannabinoids and medications with sympathomimetic effects (e.g., tachycardia, hypertension), central nervous system depressants (e.g., drowsiness, ataxia), and anticholinergics (e.g., tachycardia and somnolence). Although further studies are still pending, there is currently clinical evidence supporting drug interactions with cannabinoids, requiring doctors to evaluate the risk of drug combinations with cannabinoids and vice versa. The tables provided here were designed to facilitate the identification of biorelevant interactions that may compromise therapeutic efficacy and toxicity.

## 1. Introduction

Despite all of the possible health benefits that some scientific evidence claims it provides, the use of cannabinoids is restricted to well-identified situations and is mainly applied in cases of previous refractive therapies. Current data supports the use of cannabinoids for only a limited number of conditions, such as chemotherapy-induced nausea and vomiting, specific pain and spasticity syndromes, and certain forms of childhood epilepsy.

The most commonly prescribed drugs for the situations described above, which include analgesic, psychotropic, and cardiovascular medications, have been proven to cause potential adverse drug interactions, including with various available cannabinoids. Thus, physicians prescribing cannabinoids need to weigh the potential harms vs. perceived benefits carefully [1].

To date, only a small number of cannabinoid medicines have been approved. For instance, recently, the U.S. Food and Drug Administration (FDA) approved a drug that contains 100 mg/mL of cannabidiol (CBD) as an oral solution for the treatment of seizures associated with tuberous sclerosis complex (TSC) in patients one year of age or older. The drug was previously approved for the treatment of seizures associated with two rare and severe forms of epilepsy: Lennox–Gastaut syndrome (LGS) and Dravet syndrome (DS). This is the only FDA-approved drug that contains a purified drug substance derived from cannabis.

Previously, other drugs were approved by the FDA and also by the European Medicines Agency (EMA), such as, for example, the cannabis-related drug products dronabinol (THC; a natural cannabinoid) and nabilone (a semi-synthetic derivative of THC).

In this work, we chose to discuss the most frequent conditions and/or symptoms associated with the possible use of cannabinoids to improve their outcomes. For instance, in patients with autism spectrum disorder, the use of these drugs can be applied “off-label”. In our clinical approach, we discuss the effectiveness of these compounds, as more information is still needed to ensure a better outcome when they are added to conventional therapies. 

The use of cannabinoids in combination with other prescribed therapies can generate different types of pharmacological interactions, which is a common situation. The main objective of the present work is to alert the reader to clinically relevant interactions between cannabinoids and other medications that may impair the effectiveness and/or safety of therapy, in the hope of promoting maximum benefits and a better quality of life for patients with minimum associated risks. For their long-term use, more information is still required about isoenzyme modulation during the concomitant ingestion of these medications. In line with this concern, this brief work will contribute to improving the awareness surrounding the effects of these prescriptions, which implies a better quality of therapeutic intervention, ensuring greater safety when cannabinoids are co-administered with other medicines.

Recently (2023), a free web-based platform was developed to screen for potential drug–drug interactions, namely the CANNabinoid Drug Interaction Review, which is also a good tool for predicting these potential events [2].

In general, cannabinoids, such as Δ-9-tetrahydrocannabinol (THC) and CBD, can interact with other medications through various mechanisms. They can alter the pharmacokinetics of other drugs, particularly through interactions with the cytochrome P450 (CYP450) enzyme system, leading to increased or decreased levels of the drug in the bloodstream. Additionally, they can enhance or reduce the effects of other drugs, such as antiseizure and pain medications. It is critical to consult a healthcare professional before using cannabinoids in conjunction with other medications to avoid potential negative events [3]. These compounds, previously isolated from the cannabis plant, interact with the human endocannabinoid system. When taken in combination with other medications, they can produce a range of effects, both beneficial and adverse. Briefly, the most notable interactions include the following:

1. Enhanced or reduced drug efficacy: Cannabinoids can affect the metabolism of other drugs and alter their effectiveness. For example, THC can increase the effects of blood-thinning drugs, leading to an increased risk of bleeding.
2. Interactions with hepatic metabolism: Many drugs are processed by the liver, and cannabinoids can increase the rate at which they are metabolized, leading to reduced efficacy.
3. Adverse side effects: The combination of cannabinoids with certain drugs can lead to an increased risk of side effects, such as dizziness, fatigue, or nausea.
4. Increased sedation: Some drugs, such as opioids, can cause increased drowsiness when taken with cannabinoids, affecting the patient’s ability to perform tasks such as driving or operating heavy machinery.

In an overall approach, drug interactions correspond to a change, which is quantifiable according to the magnitude or duration of effects related to the simultaneous or previous administration of other drugs, the patient’s diet, or the pathophysiological conditions of the patient. Generally, drug interactions due to pharmacodynamic mechanisms are less common, and those reported in the literature are explained by a pharmacokinetic pathway mediated by CYP450 enzymes, P-glycoprotein (P-gp), or other drug transporters. For cannabinoids, the data indicates the following:

- THC competitively inhibits the CYP1A2, CYP2B6, CYP2C9, and CYP2D6 enzymes;
- CBD competitively inhibits the CYP3A4, CYP2B6, CYP2C9, CYP2D6, and CYP2E1 enzymes;
- Cannabinol (CBN) competitively inhibits the CYP2B6, CYP2C9, and CYP2E1 enzymes [4,5,6].

Absorption, distribution, and elimination are important phenomena that determine drug disposition on the human body but that can easily suffer changes. For example, the rate and extent of absorption can be affected by the induction or inhibition of transporter proteins in the gut, chelation phenomena, changes in gastric pH, or changes in gastrointestinal motility. The association of various food products consumed by users, or even the usual consumption of fruit and vegetable juices, protein concentrates, and/or lipids, as well as dietary supplements that include, in addition to proteins, vitamins, minerals, and medicinal plants (vegetable drugs/medicinal plants), can lead to drug interactions [7] beyond those that we delve into in more detail here.

In patients with epilepsy who are on a ketogenic diet, several preliminary aspects must be evaluated, namely the absorption and distribution conditions of the different drugs involved in therapeutic protocols, as well as the best time for their administration in light of their internal needs and the environment in which they will be ingested (fasting, before or after meals, in relation to the lipid and/or protein content of the diet, more acidic or more alkaline pH levels, etc.).

Before discussing the more specific information, it is important to point out that all cannabinoids interact with CYP2C19, and their main metabolic pathways include the CYP2C9 and CYP3A4 isoenzymes. Although for the latter polymorphisms may, in most individuals, not be problematic, the same is not linear regarding the CYP2C9 isoenzyme. This is due to the possible existence of polymorphisms or even silenced alleles that can lead, for example, to therapeutic failures and interactions with other drugs [2].

When a therapeutic treatment is made with a vegetal drug, for instance the standardized cannabis flower used as a plant drug, it contains, in addition to cannabinoids, other types of compounds such as terpenes and flavonoids. Therefore, it should be noted that most of these structures are also CYP3A4 inhibitors. Flavonoids from other plant matrices such as diosmin and hesperidin (both venotropics), or those found in extracts from *Ruscus aculeatus* L. (also a venotropic drug) as well as from *Serenoa repens* (used in the treatment of benign prostatic hyperplasia), generate similar interactions at the level of that isoenzyme. Among foods, grapefruit juice (often referred to in medicine leaflets) contains furanocoumarins, which can compromise pharmacological treatments with cannabinoids by inhibiting that isoenzyme [7].

For example, in combination with cannabis-derived compounds, the antifungal ketoconazole, a CYP3A4 inhibitor, has been reported to increase the peak concentration (Cmax) of THC and its area under the plasma concentration vs. time curve (AUC) by 1.2 and 1.8 times, respectively, with even more significant increases in the concentration of THC metabolites [8]. Other CYP3A4 inhibitors include, for instance, clarithromycin, erythromycin, cyclosporine, verapamil, itraconazole, voriconazole, and boceprevir, which may produce similar increases in THC concentrations [9].

On the other hand, one study revealed that rifampicin, a CYP3A4 inducer, reduced plasma THC levels by approximately 20–40%. However, ketoconazole, a substrate of CYP3A4 and CYP2C19, increased CBD plasma concentrations by about 2 times, while rifampicin reduced those levels by 50% to 60%. Other CYP3A4 inhibitors and inducers are likely to have a similar effect on plasma CBD concentrations if co-administered with it. However, omeprazole, which is a modest CYP2C19 inhibitor, did not change the plasma concentrations of CBD [6]. Similar situations are of special concern in cancer patients. The authors mention several aspects to be considered in these cases [9]. If the patients have a compromised liver, it is even more problematic.

Although not part of the scope of the present review, the consumption of recreational cannabis can still affect the effectiveness of some drugs, but despite everything, some in vitro studies indicate that THC and CBD have a limited capacity to inhibit the activity of CYP450 enzymes. From our modest point of view, the chronic use of cannabinoids could result, as happens with alcohol in alcoholic people, in a different outcome for the expected interactions. This means that with acute use, inhibition will be the expected effect, but when the person takes cannabinoids chronically, the effect on CYP 3A4 could be different and result, for instance, in an induction of this isoenzyme. In our patients, we verified this effect. However, the number of patients we studied is low and the data from other sources are still scarce. Despite this, an increased response to warfarin was observed in one patient who smoked four to five joints per week [10]. Also, smoking marijuana, but not consuming it orally, has been shown to increase the metabolism of theophylline and chlorpromazine, with a reduction of about 50% in their plasma concentrations. This could be due to CYP1A2 induction when smoking more than two joints per week. In fact, hemostasis needs a delicate balance between coagulation and the fibrinolytic system to achieve a normal blood circulation. Some authors highlight the links connecting cannabinoid actions with blood coagulation abnormalities, suggesting crosstalk between the coagulation system and the endogenous cannabinoid system [11].

In the meantime, pending further studies, patients taking THC should be advised to avoid medications that alter CYP3A4 and CYP2C9 activity, such as amiodarone, cimetidine, cotrimoxazole, fluoxetine, fluvoxamine, fluconazole, metronidazole, and voriconazole, which may inhibit these isoenzymes.

The tables throughout the following sections establish relationships between the various possibilities of interactions with cannabinoids and medications used for selected symptoms of different pathologies that are currently recognized as being improved with the prescription of cannabinoids. They provide highlights to assess the expected impacts of these associations in the long term, prior to the monitoring processes.

The assessment of potential interactions must be carried out in both directions: that is, both the effects of cannabinoids on other drugs and the effects of other drugs on cannabinoids or their derivatives should be studied.

To achieve successful and safe therapy, prior assessment of the impacts of any interactions that may occur is recommended, especially in personalized clinical cases. As an effective guide for clinical practice, this work summarizes the available data (clinical and non-clinical) that currently scientifically validate the pharmacological interactions between cannabinoids and other active molecules and vice versa. Despite the scientific and clinical evidence, the assessment of plasma concentrations of cannabinoids, as well as the other drugs with which they are co-administered, should be made a standard current practice to achieve greater effectiveness in personalized treatments.

To avoid all of the above-cited possible situations, which are probable to compromise the well-being of the patient, it is crucial to talk with a healthcare provider before using any medication in combination with cannabinoids, as they can advise on the potential risks and benefits. They may also suggest alternative treatments or adjust the dose of the medication to minimize any potential interactions. This work compiles, for the first time, the available data, our personal experience with patients, and practical suggestions that can be used as a clinical guide for this adjuvant therapeutic treatment.

## 2. Results

The information collected and integrated here in the tables includes the possibility of using cannabinoids to treat symptoms associated with the chronic conditions for which they are validated, such as refractory epilepsy, nausea and vomiting in chemotherapy, multiple sclerosis, and chronic pain. Assessing the potential interactions in this adjuvant therapy with cannabinoids, even if used off-label, should always be carried out for the benefit of patients.

### 2.1. Epilepsy

The pharmacological interactions of cannabinoids in the treatment of drug-resistant epilepsy both in adult populations and in children are potentially predictable. However, many gaps remain without information and future clinical trials should be considered.

Regarding the pediatric population, CBD was approved in 2018 in the USA and in 2019 in the European Union for the treatment of children aged at least two years old and diagnosed with Lennox–Gastaut syndrome or Dravet syndrome. The recommended starting dose is 2.5 mg/kg twice daily for one week, followed by an initial maintenance dose of 5 mg/kg twice daily (10 mg/kg/day). However, a slower dose escalation may be warranted, taking 3–4 weeks to reach 10 mg/kg/day. After evaluating the patient’s response, clinicians may increase the dose by 2.5 to 5 mg/kg twice daily in weekly increments as needed to achieve clinical effects. One of the drugs approved and currently in the market is a liquid formulation of CBD (1000 mg/mL) in sesame oil (flavored). It is indicated in combination with clobazam to treat patients from the age of two with Lennox–Gastaut syndrome or Dravet syndrome. It is also prescribed to treat tuberous sclerosis complex with other epilepsy treatments in patients aged two years and older. These are rare types of epilepsy that start in childhood and can continue into adulthood. Symptoms of these conditions include various types of seizures, learning difficulties, and behavioral problems. These conditions are rare and this drug has been designated as an “orphan medicine” by the EMA.

A double-blind randomized clinical trial that included 120 children and young adults with Dravet syndrome and drug-resistant seizures demonstrated that oral administration of CBD (20 mg/kg/day) concomitantly with already-instituted pharmacotherapy decreased the frequency of seizures (vs. a placebo), but increased the incidence of adverse effects (diarrhea, vomiting, fatigue, pyrexia, drowsiness, and changes in liver function) [4]. The same study highlighted that clobazam (65%), valproic acid (VPA)/valproate (59%), stiripentol (42%), levetiracetam (28%), and topiramate (26%) were the most frequently co-administered drugs. The adverse effect of drowsiness was frequent (36% in the CBD group and 10% in the placebo group). Among the 22 patients enrolled in the CBD group, 18 were also undergoing treatment with clobazam. The CBD dose was reduced in 10 patients, and adverse events were completely reversed in 8 of them. Transaminase levels were approximately three-fold above the upper limit of the normal range and led to the withdrawal of three patients from the CBD group and one from the placebo group. Overall, elevated levels of aminotransferases occurred in 12 patients in the CBD group (vs. 1 in the placebo group), all of whom were also taking VPA/valproate. The authors do not speculate on the hepatotoxicity of this medication, but the inhibition of CYP3A4 by CBD may be the reason for its high plasma concentration.

Other clinical pharmacokinetic studies have been performed with pediatric patients to understand the impact of adding CBD when co-administered with various anticonvulsant medications (e.g., carbamazepine, topiramate, and phenytoin). CBD is metabolized by CYP2C19 and CYP3A, which are either induced (e.g., VPA/valproate) or inhibited by them. This cannabinoid is also described as an inhibitor of the CYP3A4 and CYP2C family. In these associations, the consequent impact on clinical responses can be unpredictable. In one clinical trial (NCT02324673), 61 pediatric patients (1–17 years old) with drug-resistant epilepsy were enrolled and administered CBD as an add-on to their therapy [11]. This clinical trial aimed to evaluate the pharmacokinetics and short-term tolerability of CBD, determining its plasma concentrations as well as the concentration of its active metabolite, 7-hydroxyCBD (7-OH CBD). The study showed that the systemic exposure to CBD increased linearly with the increment of the administered dose. No statistically significant differences were detected between children and adolescents. However, the influence of clobazam on the AUC and Cmax of the CBD-treated patients was evident. Thus, the patients treated with CBD (40 mg/kg/day) and clobazam had a mean value of systemic exposure 10 times higher than that of the patients who did not receive clobazam (AUC: 3130 ng·h/mL vs. 1245 ng·h/ mL), while the Cmax was approximately 2.4 times higher. Similarly, a trend towards increased 7-OH CBD concentrations were observed with clobazam administration. Furthermore, CBD appeared to increase clobazam and norclobazam (N-CLB) concentrations in a dose-dependent manner: on day 10, the mean plasma concentrations of clobazam increased 1.7- and 2.2-fold in patients co-administered with CBD (40 mg/Kg/day) compared to those treated with 10 mg/kg/day and 20 mg/kg/day (857 ng/mL vs. 500 ng/mL and 390 ng/mL), respectively. Similarly, the mean N-CLB plasma concentrations were also higher in patients treated with this compound at 40 mg/kg/day compared to those treated with 20 mg/kg/day and 10 mg/kg/day (6111 ng/mL vs. 4750 ng/ mL and 3224 ng/mL, respectively).

The bidirectional interaction between these two drugs is also evident in children and adolescents, because both are substrates for CYP3A4. The significant inter-individual variability observed in the study led the authors to recommend the monitoring of serum concentrations of both drugs, together with their clinical monitoring.

These results were corroborated by another clinical trial evaluating the pharmacokinetics of CBD in children with Dravet syndrome aged between 4 and 10 years [12]. In addition to linear pharmacokinetics, a pharmacokinetic interaction with clobazam at the three studied doses of CBD (5, 10, and 20 mg/kg/day) was characterized by increased plasma exposure of N-CLB, which may contribute to antiseizure therapeutic effects and adverse effects (sedation, fatigue), even though additional sedation was not reported in this study. Interestingly, the increase in N-CLB was not observed in the presence of stiripentol, suggesting that stiripentol greatly inhibited CYP2C19, although additional evidence is needed as the sample size in the study was limited to four patients.

A larger trial was carried out to help understand the mechanistic target of the rapamycin inhibitors everolimus and sirolimus, which exhibit activity against epilepsy and multiple manifestations of tuberous sclerosis complex and are also approved for the treatment of astrocytomas, angiomyolipomas, and lymphangioleiomyomatosis. However, there is once again a lack of information on drug interactions between the mechanistic target of rapamycin inhibitors and, for example, CBD in clinical practice. The authors reviewed specially chosen patients with tuberous sclerosis complex undergoing treatment with a mechanistic target of a rapamycin inhibitor (everolimus, sirolimus) and CBD. Clinical information, the mechanistic target of the rapamycin inhibitor, the CBD dosage, concomitant anticonvulsant medications, as well as laboratory and adverse events were reviewed before and after the initiation of this cannabinoid. A total of 25 patients were treated and the mechanistic targets of the rapamycin inhibitors (18 everolimus, 7 sirolimus) and CBD were evaluated. All mechanistic targets of rapamycin inhibitor levels were determined at the trough (low point of serum concentration) just before the next dose. Levels were significantly higher in 76% of patients after CBD treatment (*p* = 0.0003). The median change from baseline was +9.8 ng/mL for everolimus and +5.1 ng/mL for sirolimus. Around 40% of the patients presented with side effects, with diarrhea being the most common (three patients). No severe adverse events happened during the treatment period. Important data include the fact that CBD increased the serum levels of everolimus and/or sirolimus. Some patients experienced doubling or tripling of their mechanistic target of rapamycin inhibitor trough after CBD was administered. In some cases, clinical toxicity was observed, as well as laboratory anomalies. Awareness of this interaction can lead clinicians to evaluate serum levels and other laboratory studies on drug safety more closely, and thereby avoid potentially significant adverse effects. In patients known to be predisposed to mechanistic target of rapamycin inhibitor toxicity, preventive reductions in the dose may be warranted upon the initiation of CBD [13]. 

Table 1 displays the most common drugs used in the clinical management of epilepsy and the possible outcomes expected when both cannabinoids, CBD and THC, are concomitantly used.

### 2.2. Autism Spectrum Disorder 

Today, there are more and more children, and even adults, diagnosed with autism spectrum disorder. In the United States, for instance, in 2023, data published by the Autism and Developmental Disabilities Monitoring Network revealed an increased prevalence of 1 in 36 children (aged 8 years) diagnosed in 2020. For the first time, the higher prevalence rates were among Black non-Hispanic, Hispanic, and Asian or Pacific Islander children compared to White non-Hispanic children. In this study, the authors also found that males showed a higher prevalence compared with females regardless of their intellectual disability status (https://www.cdc.gov/ncbddd/autism/data.html; assessed on 30 January 2024).

The drugs used to treat autism spectrum disorder are scarce and comprise the first-line stimulant drugs methylphenidate and lisdexamfetamine, followed by the second-line non-stimulant atomoxetine. The main pharmacological effects of these drugs include the inhibition of dopamine and noradrenaline transporters, increasing the concentration of these neurotransmitters in the synaptic clefts. In this way, there is a decrease in the characteristic symptoms of the disorder, improving the quality of life of the patients. These drugs are liable to suffer and/or generate interactions, requiring a careful evaluation during the clinical process. In the case of methylphenidate, interactions via CYP450 enzymes are not as problematic since it is not metabolized through this mechanism. However, with, for example, risperidone or lisdexamfetamine, whose metabolic pathways use CYP2D6, an interaction with CBD is expected to occur as it inhibits CYP2D6. Therefore, there is an increase in the plasma concentrations of risperidone and lisdexamfetamine [14].

Meanwhile, the FDA has approved the application of oral risperidone (as tablets, an oral solution, and M-TABs) for various purposes, which include the treatment of irritability associated with autism (in children aged 5 years and older). 

All antipsychotics exhibit some degree of antagonism at D2 receptors. For instance, first-generation antipsychotics induce sixty to eighty percent D2 occupancy, and second-generation antipsychotics, such as risperidone, can induce a D2 blockade. However, this effect is mostly induced by blocked serotonin receptors such as 5HT2A. This last group has a weak bond to D2 receptors and can be dissociated easily, which decreases extrapyramidal symptoms (EPSs), but they are 5HT1A receptor agonists. Risperidone produces serotonin and norepinephrine reuptake inhibition, resulting in the antidepressant effect. Moreover, the drug does not cause anticholinergic effects; this is good for patients especially elderly people with dementia [15]. All of this evidence suggests that care is required when other drugs are associated with treatments, for example CBD, which targets the same receptors. In addition, CBD induces anxiolytic and antiseizure effects through the activation of 5-HT1A receptors (receptors coupled to Gi/o proteins that induce inhibitory effects). This interaction in the human brain is unknown. However, the fact that risperidone and CBD can compete for the same receptors needs to be evaluated further with robust clinical trials. The data available give experimental evidence of a decline in 5-HT1A receptor binding in the brains of patients with epilepsy.

### 2.3. Nausea and Vomiting during Cancer Treatments

Emerging evidence suggests positive results from the use of cannabinoids associated with cancer treatments. Some authors explore the possibility of therapeutic synergy [16], but most of the time, the assessment of possible drug–drug interactions that could compromise treatment efficacy is not discussed. Specific clinical trials that could validate these possibilities are scarce and most of them include mixtures with different proportions of CBD and THC. The objective is not to improve chemotherapy itself, but only to alleviate symptoms inherent to the cancer pathology, such as insomnia, nausea, vomiting, and pain.

Due to the lack of information, as was described above for the other pathologies where cannabinoids can be co-administered with other drugs, possible interactions will also be discussed for cancer treatments. For instance, there are several antitumor drugs that use the same metabolic pathways (CYP3A4), and the concomitant intake of cannabinoids, cannabis flower extracts, and/or drugs containing cannabinoids may compromise the efficacy and safety of therapeutic protocols (e.g., anastrozole, imatinib, paclitaxel, sunitinib, tamoxifen, trabectedin, etc.; see Table 2). 

The results of two studies demonstrated that tamoxifen and several other estrogen receptor modulators can act as inverse agonists at CB1 and CB2 receptors, resulting in interactions with possible clinical consequences. Furthermore, cannabinoids can interact with other drugs and targeted therapies used in this type of cancer treatment [17].

However, there is still a gap to fill in relation to assessing the risk of potential interactions between the different medicines used in this context. Table 2 identifies the most used chemotherapy drugs and the possible changes in plasma concentrations induced by cannabinoids.

Other medications associated with therapeutic protocols (e.g., corticosteroids, analgesics, anxiolytics, and antidepressants) that induce or inhibit these pathways can also increase this bias. In an ideal treatment, each patient should have their plasma concentrations of cannabinoids and antitumor medications carefully evaluated over time to avoid risks of failure or side effects.

For bone-only metastasis, treatment begins with hormonal therapy. The phase I, multicenter, open-label, fixed-sequence study (NCT02688088) was conducted in patients treated with advanced and/or metastatic cancer drugs such as abemaciclib, palbociclib, and ribociclib. Specifically, abemaciclib, which is metabolized via the CYP3A4 pathway, was tested with rifampicin (a strong CYP3A inducer) and clarithromycin (a strong CYP3A inhibitor). Enrolled patients were over 18 years of age, had an Eastern Cooperative Oncology Group (ECOG) score between 0 and 2, and had adequate organ function. Ninety-one percent of them (44) were Caucasian, with a median age of 60 years. patients who had previously undergone surgery were excluded. This situation may disturb drug absorption or cause emesis and PK of the drug. The validated Cooperstown 5 + 1 cocktail was used in this study. The medications included were 0.2 mg of midazolam (CYP3A4), 10 mg of S-warfarin (CYP2C9), 30 mg of dextromethorphan (CYP2D6), and 100 mg of caffeine (CYP1A2), administered orally in a single dose or over two occasions. This methodology has been validated to study drug interactions in humans (medicines that have the ability to inhibit or induce cytochrome p450 enzymes). 

**Table 1:** Clinical evidence of the potential of CBD developing pharmacokinetic/pharmacodynamic interactions with antiseizure drugs (ASDs). Highlighted in green are the clinically accepted interactions; highlighted in yellow are the interactions that have scarcely been investigated in clinical practice but have a high potential in theory to compromise ASD efficacy and safety.

Antiseizure Drug	Evidence Type *	Pharmacokinetic Interactions			Pharmacodynamic Interactions			
		CBD’s Effect on the ASD	ASD’s Effect on the CBD	Mechanism of Interaction	Therapeutic Effects	Adverse Effects	Mechanism of Interaction	Clinical Recommendations
Valproic acid (VPA)	POCS [11,12,18]	↔ Cp	NR	CBD inhibits UGT1A9/2B7, which metabolizes VPA	↑ in preclinical animal models	↑ transaminase levels	Interactions at the mitochondrial level	Monitoring of transaminase levels. ↓ VPA dose or withdrawal of CBD ** [19].
	RCT [20,21,22]	↔ Cp, ↓ plasma Cmax and AUC of VPA ↔ LPP	↔ [CBD], ↑ [7-OH-CBD], ↔ LPP	NR	[7-OH-CBD] ↑ but not in a clinically relevant manner	NR	NR	
Brivaracetam	POCS	↑ Cp, > MT	NR	CBD inhibits CYP2C19, which metabolizes the ASD	NR	NR	-	↓ brivaracetam dose.
Carbamazepine (CBZ)	POCS	↔ Cp	NR	CBZ induces CYP3A4 and CYP2C19, with potential ↓ [CBD]	NA	NA	-	↑ CBD dose or ↓ CBZ dose. ASD therapeutic monitoring is advisable.
Clobazam (CLB)	Cohort observational study	↑ [N-CLB]	↑ [CBD], ↑ [7-OH-CBD]	CBD inhibits CYP2C19, responsible for N-CLB inactivation; CLB inhibits UGTs and CYPs and ↑ [7-OH-CBD]	Potentiated due to the interaction	Drowsiness, sedation, and lethargy (related to [N-CLB])	GABA_A_-R	ASD monitoring is strongly recommended. Transaminases and bilirubin must be monitored **. ↓ CLB dose or CLB withdrawal [19].
	RCT	↑ [N-CLB]	↑ [7-OH-CBD]	-	-	-	-	
Clonazepam	POCS	↔	NR	NR	-	-	-	No clinical evidence of interaction.
Eslicarbazepine (active metabolite of the prodrug eslicarbazepine acetate)	POCS	↑ Cp linearly as CBD dose ↑; ↔ MT	NR	The excipient sesamine inhibits eslicarbazepine glucuronidation	No clinically relevant changes	No clinically relevant changes	-	More studies are required. Careful monitoring and reporting of interactions are required.
Ethosuximide (ETX)	POCS	↔	NR	ETX is metabolized by CYP3A4, which is inhibited by CBD	-	-	-	No clinical evidence, but ↓ ETX dose may be considered.
Felbamate (FBM)		↑ Cp	↓↑ Cp	FLB induces CYP3A4 and inhibits CYP2C19, which metabolize CBD; CBD inhibits CYP3A4, which is responsible for FLB metabolism				CBD therapeutic monitoring is strongly recommended. ↓ FLB dose.
Fenfluramine	Unpublished data on file (Zogenix)	↔	↔	-	NR	NR	NR	No clinical evidence of interaction.
Lacosamide	POCS	↔ Cp	↑ [CBD] preclinically (animal evidence)	Lacosamide inhibits CYP2C19, CYP3A4, and CYP2C19	↔	↔	-	CBD should ideally be monitored.
Lamotrigine (LTG)	POCS	↔ Cp	Mouse model: ↔	CBD inhibits UGT1A4 and UGT2B7; Cp of LTG effect did not exhibit a significant change	Mouse model: ↔			No clinical evidence of interaction.
Levetiracetam	RCT	↔ Cp	NR	NR	NR	NR	NR	No clinical evidence of interaction.
Midazolam		↑ active metabolite (1-OH-midazolam)	NR	NR				Midazolam should be monitored.
Oxcarbazepine (OXC)	POCS	↔ Cp, ↑ Cp (preclinical mouse model)	Mouse model: OXC ↑ uptake of CBD to the brain, CBD Cp ↓ because OXC induces CYP3A4	Mouse model: CBD inhibits UGTs that conjugate the active metabolite of OXC	NR in humans Mouse model: CBD increases the therapeutic effect of OXC	-	-	More clinical studies are required. Careful monitoring and reporting of interactions are required.
Perampanel (PER)	POCS	↔ Cp, but in theory, Cp can be increased	NR	CBD inhibits CYP3A4, which metabolizes PER	NR	NR	-	Therapeutic drug monitoring is advisable because PER has a narrow MT [10].
Phenobarbital	POCS	↑ Cp	Phenobarbital induces CYP3A4 and CYP2C19, ↑ [CBD]	CBD inhibits CYP2C8/9 and CYP2C19, which metabolizes phenobarbital	Preclinical animal studies evidence no interactions	NR	-	↑ CBD dose or ↓ phenobarbital dose.
Phenytoin (PHT)	POCS	↔ Cp	NR	CBD inhibits CYP2C19, which metabolizes PHT	NR	NR	-	PHT has a narrow MT, so PHT therapeutic monitoring is strongly recommended.
Primidone		↓ Cp						
Rufinamide	POCS [23]	↑ Cp linearly with CBD dose, ↔ MT	Inhibition of carboxyl-esterases by sesamine		NR	Not observed	NR	Changes are not clinically relevant.
Sirolimus	ROCS [13,24]	↑ Cp (2–3 fold)	NR	CBD inhibits CYP3A4, which metabolizes mTOR inhibitors	NR	NR	-	Therapeutic drug monitoring is advisable for mTOR inhibitors.
Everolimus	Case report [24]	↑↑ Cp			-	-	-	
Stiripentol (STP)	RCT	↑ Cp [12,18], ↔ Cp [22]	↑ [CBD], ↓ [7-OH-CBD], ↓ [7-COOH-CBD] [12], ↔ [N-CLB] when CLB is co-administered with CBD	STP Cp ↑ because CBD inhibits CYP2C19 [12]; STP inhibits CYP3A4 and CYP2C19, decreasing CBD Cp	NR	NR	NR	Therapeutic drug monitoring is advisable because STP has narrow MT. Clinical relevance is scarce, requiring more studies.
Topiramate (TPM)	RCT [22]	↔ Cp	↑ Cp; mouse model: CBD Cp ↑ in plasma and the brain [25]	TPM inhibits CYP2C19 and induces CYP3A4 [25,26]	Mouse model: CBD activity ↑ [25]			Therapeutic monitoring of TPM and its side effects are strongly recommended [27].
	POCS [28]	↑ Cp	Higher-power test of POCS justifies differences to RCT		No clinically relevant changes [23]	-	-	
Vigabatrine	POCS [10,23]	↔ Cp	NA	-	NR	NR	-	No clinical evidence of interaction.
Zonisamide	POCS [23,28]	↑ Cp linearly as CBD dose ↑, ↔ MT	↑ [CBD], ↑ [7-OH-CBD] [19]	CBD inhibits CYP3A4, which metabolizes zonisamide	↔	NR	-	No clinical evidence of interaction.

* POCS, prospective observational clinical study; RCT, randomized clinical trial; ROCS, retrospective observational cohort study. ** Transaminases > 3× ULN and bilirubin > 2× ULN; or Transaminases > 5× ULN [5]. >, superior; <, lower; ↑, increased; ↓, decreased; ↔, variability; Cp, plasma concentration; GABA_A_R, type A receptor of gamma-aminobutyric acid; mTOR, mechanistic target of rapamycin; NR, not reported; PPB, plasma protein binding; TR, therapeutic range; ULN, upper limit of normal.

Among these medications, the information obtained with caffeine and paraxanthine (metabolite) was not valid due to possible biases associated with dietary consumption. The main conclusion shown by the authors was the absence of clinically relevant changes in the pharmacokinetics of CYP1A2, CYP2C9, CYP2D6, or CYP3A drug substrates when co-administered with multiple doses of abemaciclib. These relevant data should be replicated for the most relevant drugs, as they suggest that the mechanisms of CYP negative regulation in vitro need to be improved and better understood [29]. Although more research is needed, specifically for the topic we are writing about, these data are relevant. The crucial dose of cannabinoids that is recommended for its co-administration with medications commonly prescribed in different types of cancer is still unknown, leaving doctors waiting for the best final outcomes of this co-therapy.

One prospective randomized double-blind study carried out to evaluate the efficacy of nabilone compared to prochlorperazine involved eighty patients undergoing chemotherapy, and most of them were receiving cisplatin, a medication that produces intense nausea and vomiting. During two consecutive treatment cycles, the enrolled patients received either nabilone or prochlorperazine with identical chemotherapy treatments. Prochlorperazine is used to treat severe nausea and vomiting. In this study, the adverse effects were hypotension and lethargy, which were more pronounced with nabilone. Sixty patients (75%) reported that nabilone was more effective than prochlorperazine in relieving the evaluated symptoms. Among these patients, 46 required additional chemotherapy and continued to take nabilone as their anti-emetic of choice [30].

Another investigation evaluated the impact of nabilone in patients receiving chemotherapy. On the first day, they were treated with cyclophosphamide (CTX), adriamycin (ADR), and etoposide (VP 16); on the second day, they were treated with vincristine; and they were treated with methotrexate on the tenth day, followed by folinic acid rescue. The patients on nabilone experienced nausea, retching, and vomiting but with a lower symptom score. No patients on nabilone needed an extra anti-emetic. From the intake of nabilone, the adverse events were drowsiness, postural dizziness, and light-headedness. A low percentage experienced euphoria and a “high”. The erect systolic blood pressure was lower in the first day but postural hypotension was a problem in some patients. The authors concluded that nabilone showed effectiveness as an oral anti-emetic drug in the case of moderate toxic chemotherapy, but further studies should evaluate and better control these side effects [31].

The addition of CBD in treatments with paclitaxel (PAC) was also studied to understand its impact on chemotherapy-induced neuropathic pain (CIPN). Some patient withdraw their treatment due to this side effect. Paclitaxel is metabolized primarily into 6α-hydroxypaclitaxel by CYP2C8, and into two minor metabolites, 3′-p-hydroxypaclitaxel and 6α, 3′-p-dihydroxypaclitaxel, by CYP3A4. CBD was added to the treatment to prevent PAC-induced mechanical sensitivity. In this research, female C57Bl/6 mice were used. CBD did not produce a rewarding effect or affect their learning and memory. The combination of CBD and paclitaxel in this investigation induced an additive–synergistic inhibition of breast cancer cell viability. CBD could be a promising option against PAC-induced neurotoxicity mediated in part by the 5-HT1A receptor system. The data from the authors are promising; however, robust investigations are needed in humans [32].

A possible important use of CBD could be related to its association with tamoxifen treatments. Tamoxifen inhibits the action of estrogen at the target tissue level in hormone-responsive breast cancer. Despite good results, around forty percent of patients discontinue this therapy mainly due to side effects (arthralgia, hot flashes, insomnia, and mood changes). This medication is a prodrug metabolized mainly by the CYP2D6 isoenzyme into its main and most active metabolite, endoxifen. Due to its complex metabolism, tamoxifen is predisposed to drug interactions (including with medicinal plants and foods). CBD may also affect the pharmacokinetics of tamoxifen due to the inhibition of CYP2D6. To gain insights into how this process can be affected, an investigation was carried out involving a total of 35 patients to determine the pharmacokinetic interaction between CBD oil and endoxifen. The study began with the continuation of tamoxifen monotherapy for 7 days. The patients then took tamoxifen at 9 am and were hospitalized for a 24 h pharmacokinetic blood collection of the prodrug as well as the active metabolite endoxifen. The combined sublingual dose of pharmaceutical-grade CBD oil (three times a day/4-week dose) started with five drops of 10% (i.e., ≈50 mg of CBD per day), concomitantly with tamoxifen treatment. After that period, the patients were again hospitalized for blood collection and a pharmacokinetic study was carried out. The data from this study conclude, as preliminary results, that the patients’ arthralgia, hot flashes, and insomnia improved. However, 10 of the 26 patients presented toxicity related to the CBD oil. Although none of the patients quit the CBD oil treatment, they experienced side effects (grade 1) such as fatigue and dry mouth [33].

In other studies (phase I and II), researchers evaluated the influence of co-administration on the intake of 2.7 mg of delta-9-tetrahydrocannabinol (THC) and 2.5 mg of cannabidiol (CBD) from *Cannabis sativa* L. (each dose of a spray solution) in patients already being treated with temozolomide (TMZ) for glioblastomas. In the phase I study, patients received up to 12 sprays per day and complained of side effects such as fatigue, headache, vomiting, and nausea. For the three-year phase II trial (ARISTOCRAT), funded by The Brain Tumor Charity and coordinated by the Cancer Research UK Clinical Trials Unit at the University of Birmingham, more than 230 patients were recruited across the UK in early 2022. This trial was designed for patients who had contracted an aggressive glioblastoma that grew back after first-line treatment and who were sensitive to temozolomide. Among the inclusion criteria, the patients needed to be postmenopausal women according to standard clinical criteria or receiving concomitant therapy with a luteinizing hormone-releasing hormone (LHRH) agonist; they needed to also have undergone therapy with aromatase inhibitors (anastrozole, exemestane, or letrozole) as an adjuvant treatment for breast cancer, or for chemoprevention, for at least 3 weeks and no more than 2 years at the time of enrollment. In this new phase II trial (registered on 25 October 2022 and last edited on 31 August 2023), a randomized, double-blind, parallel-group, placebo-controlled study, the researchers will evaluate whether adding a CBD extract to the current standard of chemotherapy with temozolomide could offer longer life expectancies for adults diagnosed with glioblastoma recurrence after initial treatment. However, the authors do not mention the possible assessment of drug interactions that they expect to result from the data collected. This information will be relevant to propose, in the near future, better treatments with less risks using this combination of medicines.

Studies involving real-life scenarios with clinical relevance should be published because they will provide evidence that is sometimes difficult to obtain in clinical trials. Among those already published, Guedon et al. described results from an oncology day hospital with a cross-sectional study of 363 cancer patients treated with chemotherapy, where 20 consumed CBD. Among all patients, they described 90 interactions with 34 medications. The main clinical risks were central nervous system depression and hepatotoxicity. The main interactions were considered moderate and antineoplastic treatment did not appear to be at risk. Even so, they discontinued the CBD treatment because it needed consistent management. These data help in finding evidence for additional studies that will provide more robust information about these clinical drug interactions with CBD in cancer patients [34]. 

Despite the results highlighted in this text, we eagerly await data from the various phase II clinical trials, as described above, but also from the one investigating CBD for the treatment of aromatase inhibitor-associated arthralgias (ClinicalTrials.gov Identifier: NCT04754399). The estimated study completion date was November 2023, but the results are not yet available. The study design involved an oral CBD solution, which was administered twice a day, as follows: week 1: 25 mg twice daily, approximately 12 h apart, with food; week 2: 50 mg twice daily, approximately 12 h apart, with food; week 3: 75 mg twice daily, approximately 12 h apart, with food; week 4+: 100 mg twice a day, approximately 12 h apart, with food.

Meanwhile, the Food Standards Agency (FSA) recommended lowering the daily intake of CBD from 70 mg to just 10 mg.

**Table 2:** Evaluation and discussion of clinical evidence regarding potential developed interactions between cannabidiol (CBD) and drugs used to treat cancer.

Antineoplastic Drug(s)	Evidence Type	Study Type	Mechanism of Interaction	Clinical Outcomes	Notes/References
**CDK4/CDK6 Inhibitors** Abemaciclib (Palbociclib) (Ribociclib)	Clinical	Phase I clinical trial: a multicenter, open-label, fixed-sequence study conducted in patients with advanced and/or metastatic cancer.	CYP1A2, CYP2C9, CYP2D6, and CYP3A substrate drugs.	No clinically relevant changes observed in the PK of the selected CYP when co-administered with multiple doses of abemaciclib.	Participants were also asked to refrain from consuming grapefruit juice, Seville oranges, and St. John’s Wort during the study’s time frame. No recommendations were given about consuming caffeinated drinks [29]. (ClinicalTrials.gov Identifier: NCT02688088)
**Aromatase Inhibitors** Anastrozole Exemestane Letrozole (although fulvestrant belongs to the same group, it was not included in this trial)	Clinical	Phase II clinical trial investigating CBD (Epidiolex) for the treatment of aromatase inhibitor-associated arthralgias (arthralgia and breast cancer).	Evaluation of the safety and efficacy of CBD treatment in postmenopausal women with aromatase inhibitor-associated musculoskeletal symptoms (AIMSSs) due to anastrozole intake (15-week duration).	The investigators are looking to see if patients with joint pain experience an improvement with the concomitant use of CBD.	No results posted yet (end of the trial: October 2023). Although clinical evidence is scarce regarding anastrozole’s inhibition of CYP 1A2, 2C8/9, and 3A4, studying the effects of its concomitant use with CBD, as performed in this clinical trial, will provide relevant data for future prescribed treatments. (ClinicalTrials.gov Identifier: NCT04754399)
**Carboplatine** **Cisplatin**	Clinical	Prospective study investigating nabilone (a synthetic cannabinoid) as an effective anti-emetic in patients receiving cancer chemotherapy.	NR	Sixty patients (75 per cent) reported nabilone to be more effective than prochlorperazine for relieving nausea and vomiting. Of these 60 patients, 46 required further chemotherapy and continued taking nabilone as their anti-emetic of choice.	[30]
**Cyclophosphamide** (adjuvant or not with doxorubicin or a taxane)	Clinical	Comparative study investigating the anti-emetic efficacy and toxicity of nabilone in lung cancer chemotherapy.	CYP P450 isoforms, CYP2A6, 2B6, 3A4, 3A5, 2C9, 2C18, and 2C19.	Symptom scores were significantly better for patients on nabilone regarding nausea, retching, and vomiting (*p* less than 0.05); fewer subjects vomited (*p* = 0.05) and the number of vomiting episodes was lower (*p* less than 0.05); no patients on nabilone required an additional parenteral anti-emetic. More patients preferred nabilone for anti-emetic purposes (*p* less than 0.005).	Of the 34 patients that entered the study, 6 dropped out after the first course and 2 patients did not complete a course because of adverse effects, leaving 26 patients who completed the crossover study. Four of these entered the study on their first cycle of chemotherapy; they had received one prior cycle with a standard phenothiazine anti-emetic and had all experienced mild to moderate gastrointestinal toxicity and so were considered suitable for inclusion in the analysis [31].
**mTOR Inhibitors** Everolimus	Clinical	Clinical study investigating whether CBD elevates mechanistic target of rapamycin inhibitor levels in patients with tuberous sclerosis complex.	CYP3A4 metabolism. Everolimus is a substrate of CYP3A4 and PgP (phosphoglycolate phosphatase). Three monohydroxylated metabolites, two hydrolytic ring-opened products, and a phosphatidylcholine conjugate of everolimus were the six primary metabolites detected in the human blood. In vitro, everolimus competitively inhibited the metabolism of CYP3A4 and was a mixed inhibitor of the CYP2D6 substrate dextromethorphan.	CBD resulted in increased serum levels of everolimus.	[13]
**Paclitaxel**	Preclinical	Preclinical study investigating whether CBD inhibits paclitaxel-induced neuropathic pain through 5-HT1A receptors without diminishing nervous system function or chemotherapy efficacy.	Metabolism of CYP2C8/9.	Paclitaxel-induced mechanical sensitivity was prevented through the administration of CBD (2.5–10 mg·kg^−1^) in female C57Bl/6 mice. This effect was reversed via co-administration of the 5-HT1A antagonist WAY 100635, but not the CB1 antagonist SR141716 or the CB2 antagonist SR144528. The CBD produced no conditioned rewarding effects and did not affect the mice’s learning and memory.	CBD + paclitaxel combinations induced additive-to-synergistic inhibition of breast cancer cell viability [32].
**Selective Estrogen Receptor Modulators** Tamoxifen	Clinical	Clinical trial investigating CBD oil as a potential solution in cases of severe tamoxifen-related side effects.	Metabolism of CYP3A4 and 2D6.	None of the patients quit the CBD oil treatment because of side effects and sixty-nine percent of the patients wished to continue taking CBD oil after the study was finished. The authors suggested that CBD oil, with a dosage below 50 mg, does not have to be discouraged in patients using it for tamoxifen-related side effects.	The use of CBD oil allowed the authors to conclude that hot flashes and arthralgia improved by at least one grade in 6 out of 25 patients (24%) and insomnia improved by one grade in 11 out of 26 patients (42%). This is in line with the trend seen in the improvements in separate endocrine subscale items. Ten out of twenty-six patients (38%) experienced some kind of CBD-oil-related toxicity. The most frequently mentioned side effects were fatigue (*n* = 3, 12%) and dry mouth (*n* = 3, 12%) [33].
**Temozolomide**	Clinical	Phase I and II clinical trial investigating the use of the cannabis-based drug Sativex with current chemotherapy treatments in treating patients with recurrent glioblastoma.	Non-enzymatic hydrolysis of temozolomide into its active metabolite 5-(3-metiltriazeno1-il) imidazol-4-carboxamida (MTIC) at neutral pH. It is then secreted by the kidneys.	This trial was set up for patients who had an aggressive glioblastoma that had grown back after first-line treatment. They also needed to have the subtype of glioblastoma that is sensitive to temozolomide.	The metabolization was not as such related with CYP450 metabolization, so it was not as such influenced by interactions with other medications. Plasma protein binding at 1 and 4 h showed mean free fractions of radioactivity of 84%; 12–16% of drug-derived radioactivity was bound to plasma proteins; 1% of the 14C-temozolomide dose was recovered in the patients’ feces and 38% was recovered in the patients’ urine over the 360 h collection period [35].

NR, not reported.

### 2.4. Symptoms of Multiple Sclerosis and Pain

Here, only the questions related to the symptoms that could also be treated with cannabinoids will be discussed. Treatment for multiple sclerosis is tailored to the specific symptoms of the person and the stage of the disease. Interactions between cannabinoids and other centrally acting drugs will be discussed in this same section since they have similar mechanisms. 

The medications highlighted in Table 3 are currently used in a variety of conditions and the table also displays the information available regarding their concomitant use with cannabinoids. For example, CBD increases the plasma concentrations of selective serotonin reuptake inhibitors (SSRIs), tricyclic antidepressants, and antipsychotics, compromising their pharmacological effects due to its inhibitory capacity on the CYP2D6 isoform. It also interacts with monoamine oxidase inhibitors (MAOIs) such as tranylcypromine, phenelzine, and isocarboxazid, inhibiting their metabolism and consequently increasing the average residence time and half-life of antidepressants, enhancing and prolonging their adverse effects. Clinical trials exploring pharmacological interactions between cannabinoids and antidepressants are rare or virtually nonexistent [6]. However, it has been shown in animal models of depression and post-traumatic stress disorder that co-administration of CBD with the SSRI sertraline has a synergistic effect on the development of cognitive and emotional disorders (e.g., severe anxiety and aggressive behavior) [36]. In fact, sertraline is metabolized by CYP3A4, which is inhibited by CBD, leading to an increase in plasma concentrations of the drug and the development of adverse events [37]. Therefore, this pairing should be applied in clinical practice with great care.

Divergently, when administered at subtherapeutic doses concomitantly with CBD, the noradrenergic antidepressant desipramine has an antidepressant effect, suggesting the existence of a synergistic mechanism. Despite coming from preclinical studies, these results show that CBD has antidepressant effects dependent on the serotonin levels in the central nervous system.

Furthermore, for many other antidepressants, namely citalopram, paroxetine, mirtazapine, and amitriptyline metabolized by isoforms of the CYP superfamily inhibited by CBD, when used in co-administration, CBD can potentiate their adverse effects (Table 3). For example, amitriptylineis metabolized by CYP2D6, CYP2C19, CYP 2C9, CYP3A4, and CYP1A2, which are inhibited by CBD. Therefore, their co-administration may intensify adverse effects such as anticholinergic syndrome, QT interval prolongation, and drowsiness [37]. 

Although opioids are frequently prescribed to treat and manage chronic pain, data on their association with cannabinoids are scarce and clinical and real-world evidence is not robust, which is why they have not been addressed here. Upcoming high-quality controlled clinical trials should be mandatory to control the opioid-sparing effect of cannabinoids.

## 3. Discussion

Despite all of the data already available, the same key idea remains: that personalized therapy must be carefully monitored for possible interactions but also to ensure that the therapeutic doses used are at the expected plasma levels.

Regarding the possible side effects, the data collected in EudraVigilance—a European database of reports of suspected adverse drug reactions (https://www.adrreports.eu/en/index.html; https://dap.ema.europa.eu/analytics/saw.dll?portalpages; accessed on 30 December 2023)—for already-approved and prescribed medicines used in combination with cannabis compounds are still scarce. Certainly, the fact that they are prescribed less often allows doctors to better control them, especially because the stabilization of pathologies that benefit from these associations is a challenge and patient monitoring is carried out very carefully.

To this day, several institutions are aware of these drug interactions and present data and alerts on their websites to prevent them. Very briefly, for instance, the list of cannabinoid drugs (as precipitants) that affect the metabolism of other drugs (objects) provided by Pennsylvania State University, College of Medicine, Department of Pharmacology (Hershey, PA, USA) is a good example (https://sites.psu.edu/cannabinoid; assessed 30 January 2024).

In the near future, personalized assessments of a patient’s genetic sensitivity to cannabinoid intake should always be a starting point for therapy. Future research directions can also be highlighted. For example, based on feedback regarding clinical practice in palliative care, patients who use cannabinoids with opioids are initially recommended to reduce the daily dose of the latter, but after a few days, they need to return to higher levels to obtain a better improvement in pain relief. These types of situations require clinical trials, which can be observational. Those allow a better understanding of the appropriate doses to prescribe to patients, resulting in more real clinical evidence, which complements the data provided by clinical trials.

## 4. Materials and Methods

For this work, a scoping review was carried out following the evidence synthesis approach, which is defined as “the review of what is known from existing research using systematic and explicit methods in order to clarify the evidence base” [38]. This is the best way to conduct a critical review for the translation of knowledge, which is the current purpose of this work, and to ensure that decisions are based on the best available evidence. Scoping reviews are a relatively new type of evidence synthesis that is increasingly being used in biomedical research fields and, perhaps to a lesser extent, in other various research disciplines, albeit still lacking a full universal consensus about their definition, practical methodologies, and use circumstances [39]. Details of this are specified in our recent previous work [40]. Briefly, this type of review aims primarily to figure out and map the extent (depth and breadth) of scientific evidence on a particular topic, field, concept, or issue, unveil potential knowledge gaps, and possibly clarify key concepts, thus informing both research conduct and practical decision and policy making. These reviews distinguish themselves from other reviews, such as systematic reviews, by offering an extended breadth of includible literature, a more systematic and comprehensive selection of the available scientific literature, and greater flexibility in drawing conclusions and receiving criticism from scientific communities to further advance research in the tackled topic. They approach broadly diversified topics and also allow analyses of a wide range of data and topics [37,40].

As it permitted us to rely on all stages of pharmaceutical, medical, and biomedical research, including experimental, preclinical, translational, clinical, and real-world evidence phases, a scoping review was then preferable from our point of view. This is mainly due to the fact that published works on these compounds are characterized by a very rapid expansion, especially in very recent years, and thus the most integrative information could be used for analysis and discussion.

The PRISMA Extension for Scoping Reviews (PRISMA-ScR) was followed. To be part of this review, papers needed to include the selected medical conditions, cannabinoids as a co-therapy, and pharmacokinetic and/or pharmacodynamic interactions. Only peer-reviewed publications published between 2019 and 2023, written in English, involving animal data, human participants, clinical trials, and selected symptoms associated with chronic conditions, including epilepsy, ASD, cancer (nausea and pain), multiple sclerosis, and chronic pain, were included. Treatments for these purposes were checked based on the approval of the drugs used. Papers were excluded if they did not fit into the conceptual framework of the study, if they focused on substance abuse, if no drug interactions were investigated, and if they only contained in vitro/in silico evidence.

Detailed evaluations of potential interactions in adjuvant therapy with cannabinoids were provided for the selected pathologies where cannabinoids have been approved and/or can be used off-label for the benefit of the patient.

A literature search was performed on the PubMed (MEDLINE), Medscape, and Clinical Trials databases. The search terms were “cannabinoid” AND “drug interaction” OR “anticonvulsant” OR “antiseizure” OR “analgesic” OR “cancer” OR “chronic pain” OR “Autism” OR “multiple sclerosis”.

The authors screened all retrieved papers and included all original studies written in English, published as full papers or abstracts, that met the selection criteria. The selection criteria included studies with participants diagnosed with ASD treated with *C. sativa* L extracts or isolated cannabinoids, such as CBD, CBDV, THC, etc., with or without a comparison group. Due, again, to scarce data and since the reported outcomes were expected to vary, no specific outcomes were defined to facilitate a comprehensive evaluation of the available studies in this area. All potentially eligible studies were considered regardless of the study design. At the initial screening, the studies were assessed independently for potential inclusion according to their title and abstract. Following the initial screening, the full text of each eligible publication was examined, and a final decision for its inclusion was made. In addition, citations in the selected articles were reviewed for identifying supplementary eligible articles. We extracted information about each study’s design, the characteristics of its participants, the characteristics of the treatment, and the observed outcomes and adverse effects. We also collected reported data regarding ongoing studies, as retrieved from ClinicalTrials.gov. The results of this review are presented in a narrative summary and summarized in tables organized around the characteristics of the studies.

## 5. Conclusions

Although there is more and more research in this area nowadays, there are still many gaps. The data provided to healthcare professionals must be translational research that validates the evidence needed to use cannabinoids more safely. Medication reconciliation involves evaluating potential drug interactions. Prescribed medications may have drug interactions with each other or with cannabinoids, but also with the simultaneous intake of herbal medicines, over-the-counter medications, and supplements. These all contribute to altering the results of therapy due to their impacts on pharmacodynamics and pharmacokinetics. All data in this review provide, for the first time, clear indications for validated treatments for chronic medical conditions with cannabinoid-based medicines and will be useful in increasing patient safety. However, pharmacovigilance must provide notifications of side effects and/or abnormal events, information collected in the real world, for a better understanding of this process and to improve all recommendations. It is clear that clinical data are scarce and more robust information is required to allow doctors to deal with the safe and more accurate prescription of cannabinoids as a co-adjuvant therapy to supplement other treatments.

## Figures and Tables

**Table 3:** Clinical evidence regarding the potential of cannabidiol (CBD) to develop interactions with other centrally acting drugs [6,10,36,37].

Drug	Type of Interaction	Clinical Outcomes and/or Recommendations
Amitriptyline	Antidepressant metabolized by CYP2D6, CYP2C9, CYP2C19, and CYP3A4, which are inhibited by CBD.	↑ Cp ↑ adverse events
Citalopram	Antidepressant metabolized by CYP2C19 (38%), CYP2D6 (31%), and CYP3A4 (31%), which are inhibited by CBD.	↑ Cp ↑ adverse events
Desipramine	Antidepressant and moderate inhibitor of CYP3A, which metabolizes CBD.	↑ CBD Cp ↓ CBD dose should be pondered
Diazepam	Benzodiazepine metabolized by CYP2C19, which is inhibited by CBD.	↑ Diazepam Cp ↓ Diazepam dose should be pondered
Escitalopram	Antidepressant metabolized by CYP2C19, which is inhibited by CBD.	↑ Cp ↑ adverse events
Fluoxetine (FLX)	Antidepressant metabolized by CYP2C19 and CYP2D6 (31%), which are inhibited by CBD. FLX is a moderate inhibitor of CYP2C19, which metabolizes CBD.	↑ Cp of FLX and CBD ↑ adverse events ↓ doses of both drugs should be pondered
Fluvoxamine	Antidepressant metabolized by CYP1A2, which is inhibited by CBD. It is a moderate inhibitor of CYP3A4 and a strong inhibitor of CYP2C19, which metabolize CBD.	↑↓ Cp of fluvoxamine ↑ CBD Cp ↓ CBD dose should be pondered Therapeutic monitoring of fluvoxamine is recommended.
Haloperidol	Antipsychotic drug that moderately inhibits CYP2C19, which metabolizes CBD.	↑ CBD Cp ↓ CBD dose should be pondered
Imipramine	Antidepressant metabolized by CYP2C19 and CYP2D6 (31%), which is inhibited by CBD.	↑ Cp ↑ adverse events ↓ imipramine dose should be pondered
Lorazepam	Benzodiazepine drug metabolized by UGT2B7, which is inhibited by CBD.	↑ Cp ↑ somnolence adverse events ↓ lorazepam dose should be pondered
Mirtazapine	Antidepressant drug metabolized by CYP1A2, CYP2D6, and CYP3A4, which are inhibited by CBD.	↑ Cp ↑ adverse effects
Modafinil	Neurotropic drug that is a substrate and an inhibitor of CYP2C19.	↑ Cp of modafinil and CBD ↓ doses of both drugs should be pondered Monitoring each drug’s Cp is recommended to define/optimize drug posology individually.
Olanzapine	Antipsychotic drug that is a substrate of CYP1A2, which is inhibited by CBD.	↑↓ olanzapine Cp Monitoring olanzapine’s Cp is strongly recommended to define/optimize drug posology individually.
Paroxetine	Antidepressant drug metabolized by CYP2D6, which is inhibited by CBD.	↑ Cp ↑ adverse effects
Sertraline	Antidepressant drug metabolized by CYP2C9, CYP3A4, and CYP2C19, which are inhibited by CBD. It is also a moderate inhibitor of CYP2C19 and CYP3A4, which metabolize CBD.	↑ Cp of sertraline and CBD ↓ doses of both drugs should be pondered.
Trimipramine	Antidepressant drug metabolized by CYP2C19, which is inhibited by CBD.	↑ Cp ↑ adverse events ↓ trimipramine dose should be pondered

↑, increased; ↓, decreased; ↑↓, variability (i.e., enhanced or decreased); Cp, plasma concentration.

## Data Availability

Data sharing is not applicable.
